# Large-scale identification of ubiquitination sites on membrane-associated proteins in *Arabidopsis thaliana* seedlings

**DOI:** 10.1093/plphys/kiab023

**Published:** 2021-01-28

**Authors:** Lauren E Grubb, Paul Derbyshire, Katherine E Dunning, Cyril Zipfel, Frank L H Menke, Jacqueline Monaghan

**Affiliations:** 1 Department of Biology, Queen’s University, Kingston, Canada; 2 The Sainsbury Laboratory, University of East Anglia, Norwich Research Park, Norwich, UK; 3 Department of Plant and Microbial Biology, Zurich-Basel Plant Science Center, University of Zurich, Zurich, Switzerland

## Abstract

An analysis of the identification of ubiquitination sites on proteins found at the cell periphery, including over 100 protein kinases.

Dear Editor,

Protein phosphorylation and ubiquitination are two of the most frequently observed post-translational modifications in eukaryotes, regulated by thousands of protein kinases, phosphatases, E3 ubiquitin ligases, and ubiquitin proteases. Although previous studies have catalogued several ubiquitinated proteins in plants (Walton et al., 2016), few ubiquitinated membrane-localized proteins have been identified. Receptor kinases (RKs) initiate phosphorylation signal relays that regulate plant growth, development, and stress responses. While the regulatory role of phosphorylation on protein kinase function is well-documented (Couto and Zipfel, 2016), considerably less is known about the significance of ubiquitination on protein kinases, even though their turnover is critical to signaling competence and cellular homeostasis. Here, we describe the large-scale identification of ubiquitination sites on Arabidopsis (*Arabidopsis thaliana*) proteins associated with or integral to the plasma membrane, including over 100 protein kinases.

Proteins can be mono-, poly-, and/or multi-mono-ubiquitinated, each affecting protein function in different ways (Vierstra, 2012; Swatek and Komander, 2016). Dynamic interplay between phosphorylation and ubiquitination has been observed in several proteins involved in immune signaling (Mithoe and Menke, 2018), including layered post-translational regulation of the receptor-like cytoplasmic kinase (RLCK) BOTRYTIS-INDUCED KINASE1 (BIK1). BIK1 is directly phosphorylated and activated by several ligand-bound RKs (Couto and Zipfel, 2016), and can be dephosphorylated by the phosphatase PP2C38 (Couto et al., 2016). Precise control of BIK1 abundance is regulated by poly-ubiquitination by the E3 ligases PLANT U-BOX25 (PUB25) and PUB26 (Wang et al., 2018), as well as phosphorylation by CALCIUM-DEPENDENT PROTEIN KINASE28 (CPK28; Monaghan et al., 2014; Wang et al., 2018) and the mitogen-activated protein kinase kinase kinase kinase (MAP4K) SERINE/THREONINE KINASE1 (SIK1)/MAP4K4 (Zhang et al., 2018; Jiang et al., 2019). Most recently, it was shown that BIK1 is also mono-ubiquitinated by the E3 ligases RING-H2 FINGER A3A (RHA3A) and RHA3AB to regulate its activation and endocytosis (Ma et al., 2020).

Proteomics and mutagenesis approaches have resulted in the discovery of several phosphorylated residues on BIK1 (Liang and Zhou, 2018). To help us understand the role of ubiquitination on BIK1 function, we set out to identify *in vivo* ubiquitination sites on BIK1. We enriched for plasma membrane-localized BIK1 by isolating microsomal protein fractions from Col-0/*pBIK1:BIK1-HA*, *cpk28-1/pBIK1:BIK1-HA*, and *CPK28-OE1/pBIK1:BIK1-HA* genotypes, which express 100-fold higher levels of *BIK1* and differentially accumulate BIK1 protein compared to wild-type (Monaghan et al., 2014). To increase protein abundance of nonintegral proteins and allow us to potentially capture immune-induced ubiquitination, proteasomal machinery was inhibited with 50 μM MG-132 an hour before treatment with water or 1 μM elf18 (an immunogenic peptide derived from bacterial EF-Tu; Zipfel et al., 2006). Microsomal protein fractions were digested with trypsin, and anti-K-ε-GG agarose beads (Udeshi et al., 2013) were used to enrich ubiquitinated peptides by affinity binding. Ubiquitinated lysines were identified based on a shift of ∼114 Da—the mass of two glycine remnants that remain covalently bound to lysines following trypsin digestion—using liquid chromatography followed by tandem mass spectrometry (Supplementary Methods). The mass spectrometry proteomics data have been deposited to the ProteomeXchange Consortium via the PRIDE (Perez-Riverol et al., 2019) partner repository with the dataset identifier PXD021992 and 10.6019/PXD021992.

We filtered our data for peptides with the diGly ubiquitin remnant, setting a threshold Mascot ion score of >20 and required multiple spectra for each peptide. This resulted in the identification of a total of 916 ubiquitinated peptides on 450 proteins across several biological replicates with a peptide false discovery rate of 0.025 (Supplemental Table S1), and an additional 526 peptides on 398 proteins observed in single experiments (Supplemental Table S2). Included in these data were seven ubiquitinated lysines on BIK1 (Table 1 andFigure 1;Supplemental Tables S1–S2). Given our particular interest in BIK1, we manually inspected all spectra mapping to BIK1 and found an additional three sites (Figure 1;Supplemental Figure S1), altogether corroborating five of the ubiquitinated residues reported by (Ma et al., 2020) and revealing five novel ones (Figure 1). Thus, BIK1 is ubiquitinated on multiple surface-exposed lysines *in vivo*: three in the N-terminal variable domain (K31, K41, K61), seven in the canonical kinase domain (K95, K106, K155, K170, K186, K286, K337), and five in the C-terminal region (K358, K366, K369, K374, K388; Figure 1). Whether RHA3A/B and PUB25/26 compete for these sites or ubiquitinate distinct lysines remains to be tested experimentally, as does clarifying which E2 conjugating enzymes work with respective E3 ligases to catalyze these events (Turek et al., 2018). Furthermore, as the phospho-status of BIK1 has been shown to affect its regulation by both RHA3A/B and PUB25/26 (Wang et al., 2018; Ma et al., 2020), another challenge will be resolving the biochemical mechanisms underlying this interplay.

**Figure 1.**
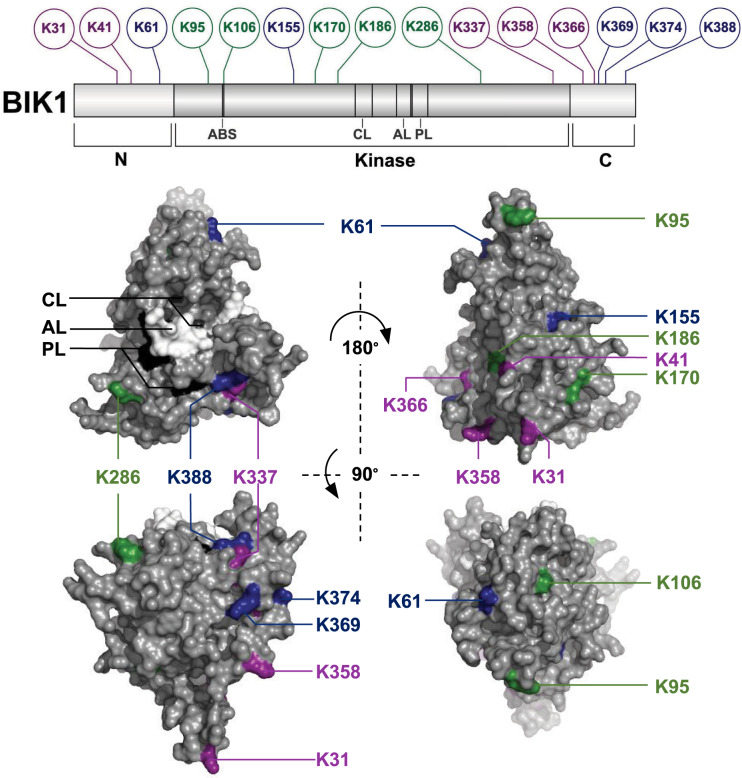
BIK1 is ubiquitinated on multiple lysines *in vivo.* A, Comparison between this study and Ma et al. (2020) indicates that BIK1 is ubiquitinated on three lysines at its amino (N) terminus, seven in its kinase domain, and five at its carboxyl (C) terminus. Ubiquitinated lysines identified in Ma et al. (2020) are shown in green, those identified in this study are shown in blue, and residues identified in both studies are in magenta. The ATP-binding site (ABS), catalytic loop (CL), activation loop (AL), and P +1 loop (PL) are indicated; the ABS is not surface-exposed, but the CL is shown in dark gray, the AL in white, and the PL in black. Although the structure of the BIK1 canonical kinase domain was recently solved (Lal et al., 2018), we modeled BIK1 in Phyre2-intensive mode (Kelley et al., 2015) in order to include the disordered N- and C-terminal ends in this surface representation in PyMol (The PyMol Molecular Graphics System, Version 2.0 Schrodinger, LLC). Phyre2-intensive modeling maximises sequence coverage and confidence to model regions for which there is no template information by an *ab initio* simplified-folding physics simulation; while 354/395 (90%) of the residues were modeled at >90% accuracy, it is likely that the model does not completely reflect the protein structure.

**Table 1.** Ubiquitinated protein kinases identified in this study. Proteins matching the gene ontology term “kinase activity” were filtered from Supplementary Tables S1 and S2 and classified based on phylogenies presented by Shiu and Bleecker (2001, 2003). Residues that are only supported by a single observation (Supplementary Table S2) are indicated by an asterisk and should be interpreted with caution. Residues that were observed only after manual inspection of mass spectra matching BIK1 are indicated with two asterisks and shown in Supplementary Figure S1

**Receptor-like protein kinases**			
**Protein family**	**Accession**	**Protein name**	**K-GG**
SD-1	AT1G11300	EGM1	K514, K527
	AT4G27300	SD1-1	K518*, K550*, K648
	AT4G21380	ARK3/RK3/SD1-8	K661
	AT1G11350	CBRLK1/RKS2	K528
	AT1G61550	S-locus lectin protein kinase family protein	K507
	AT1G11330	RDA2	K529, K542
	AT1G61380	LORE/SD1-29	K493, K506*
SD-2	AT2G19130	S-locus lectin protein kinase family protein	K498*, K591
	AT1G34300	Lectin protein kinase family protein	K489*, K710
	AT4G32300	SD2-5	K641*, K727
L-LEC	AT4G28350	LecRK-VII.2	K339
	AT3G53380	LecRK-VIII.1	K374
	AT2G37710	RLK/LecRK-IV.1	K350*, K370
C-LEC	AT1G52310	C-type lectin receptor kinase	K265*, K278*, K292
CRK/DUF26	AT1G70520	CRK2	K379*
	AT4G23180	CRK10/RLK4	K438*, K449
	AT4G23190	CRK11/RLK3	K349, K366, K368, K400*, K451
	AT4G23300	CRK22	K352, K369, K371, K381
	AT4G05200	CRK25	K448, K507
	AT4G11530	CRK34	K363, K399
	AT4G04570	CRK40	K376*, K402
URK-II	AT5G20050	URK-II family protein	K199*, K156
CrRLK1L-1	AT5G54380	THE1	K480*, K526, K534, K560*, K657*, K753*
	AT3G51550	FERONIA	K530, K534, K549, K561, K672, K759, K771, K773, K781*, K843*
	AT3G46290	HERK1	K479, K498, K501
	AT1G30570	HERK2	K518
	AT2G23200	CrRLK1L-1 family protein	K710
	AT5G38990	MDS1	K541, K554*, K646*
LRR-I	AT1G51800	IOS1	K721
	AT1G51890	LRR-Ia family protein	K543*
	AT2G37050	BSR050	K740
LRR-II	AT4G33430	SERK3/BAK1	K339*
	AT2G13800	SERK5/BAK8	K303
	AT5G10290	LRR-II family protein	K276, K314, K469
	AT5G16000	NIK1	K320*
	AT2G23950	CLERK	K317
LRR-III	AT3G17840	RLK902	K315, K336, K347
	AT1G48480	RKL1	K353, K506
	AT5G58300	LRR-III family protein	K326*
	AT2G26730	LRR-III family protein	K293, K315*, K416
	AT2G36570	PXC1	K319
	AT3G08680	LRR-III family protein	K407
	AT5G16590	LRR1	K317
STRUBBELIG-receptor	AT1G53730	SRF6	K392*
	AT3G14350	SRF7	K322
	AT4G22130	SRF8	K344*, K353
LRR-VI	AT5G63410	LRR-VI family protein	K397, K427
	AT2G02780	LRR-VI family protein	K403*
LRR-VII	AT3G28040	LRR-VIIa family protein	K742, K728
	AT1G80870	LRR-VIIa family protein	K89
LRR-VIII	AT5G49760	HPCA	K600*, K605, K625, K685*
	AT3G14840	LIK1	K666, K677, K688*, K700, K774, K793, K808, K821, K963*
LRR-IX	AT1G66150	TMK1	K640, K746
	AT2G01820	TMK3	K601, K637*, K743, K812
	AT3G23750	BARK1	K736*
LRR-X	AT5G48380	BIR1	K354, K562
	AT3G28450	BIR2	K290, K514
LRR-V	AT5G42440	LRR-Xb family protein	K109
	AT2G01820	PSKR1	K757*
LRR-XI	AT5G65700	BAM1	K785
	AT3G49670	BAM2	K781, K914
	AT1G28440	HSL1	K845, K957*
	AT1G09970	RLK7/LRR XI-23	K689, K703*, K818, K904, K966*
	AT2G33170	LRR XI family protein	K835*
	AT5G25930	LRR XI family protein	K701*, K941*
	AT1G72180	LRR XI family protein	K704*
LRR-XII	AT5G20480	EFR	K999, K1004
	AT5G46330	FLS2	K924, K940
LRR-XIII	AT1G27190	BIR3	K339
	AT1G31420	FEI1	K358
	AT4G08850	MIK2/BSR850	K770, K788, K793, K803, K818
	AT2G26330	ERECTA	K668
LRR-XV	AT3G02130	RPK2/TOAD2/CLI1	K1144
LRR-other	AT2G31880	SOBIR1/EVR	K640*
LysMa	AT3G21630	CERK1	K452
LRK10L-1a	AT1G25390	LRK10L4	K309
CRINKLY4-Like	AT5G46080	Protein kinase superfamily protein	K293
	AT3G55950	CCR3	K514
RKF3-Like	AT1G11050	Protein kinase superfamily protein	K449
WAK-Like	AT1G21250	WAK1/PRO25	K403, K425, K437
	AT1G21270	WAK2	K420, K432, K668*
	AT2G23450	WAKL family protein	K653*
Phototropin	AT3G45780	PHOT1/NPH1/RPT1	K526, K899

**Cytoplasmic protein kinases**			
**Protein family**	**Accession**	**Protein name**	**K-GG**
RLCK-V	AT3G59110	RLCK-V family protein	K206
RLCK-VII	AT2G39660	BIK1	K31*,K41, K61, K155**, K337*, K358*, K366**, K369**, K374*, K388
	AT2G17220	PBL32/KIN3	K99, K242, K347
	AT5G13160	PBS1	K204
	AT5G18610	PBL27	K201
	AT5G03320	PBL40	K115*
RLCK-VIII	AT1G06700	PTI1-1	K71
	AT2G30740	PTI1-2	K38, K74
	AT3G59350	PTI1-3	K116, K133
	AT2G47060	PTI1-4	K46, K303*
	AT3G17410	PTI1-7/CARK1	K89, K190, K299
RLCK-XII	AT4G35230	BSK1	K85
	AT4G00710	BSK3	K67, K481*
	AT5G59010	BSK5	K64*
	AT3G54030	BSK6	K65
	AT1G63500	BSK7	K68*, K105, K304*
RLCK-XV	AT1G52540	RLCK-XV family protein	K249*
PERK	AT3G24550	PERK1	K303
	AT4G32710	PERK14	K18*
CDPK	AT3G20410	CPK9	K71, K115, K427
	AT4G04720	CPK21	K84, K526*
	AT5G66210	CPK28	K25, K34, K48*, K97, K108, K206*, K217, K351, K401*, K785
MAPK	AT3G63260	MRK1/RAF48	K342
Other protein kinase	AT1G65950	Protein kinase superfamily protein	K421
	AT4G00300	Fringe-related protein	K775
	AT1G56145	LRR transmembrane protein kinase	K725
	AT3G27560	ATN1	K44*
	AT1G03740	Protein kinase superfamily protein	K56*
	AT3G25840	PRP4KA	K462*
	AT4G35500	Protein kinase superfamily protein	K247*
	AT5G05200	Protein kinase superfamily protein	K31*
	AT5G40540	Protein kinase superfamily protein	K44*
	AT5G38480	GRF3/RCI1	K52
Other kinase	AT1G12000	Phosphofructokinase family protein	K23
	AT4G21534	SPHK2	K49, K59
	AT4G09320	NDPK1	K106
	AT5G50780	AtMORC4	K736*, K766
	AT1G12330	Cyclin-dependent kinase-like protein	K184
	AT4G36080	Inositol or phosphatidylinositol kinase	K3581*
	AT1G20930	CDKB2;2	K88*
	AT5G26667	PYR6	K54*
	AT4G29130	GIN2/HXK1	K117*
	AT1G10900	Phosphatidylinositol-4-phosphate 5-kinase family protein	K28*

Analysis of gene ontology (GO) terms associated with proteins identified in the high-confidence dataset (Supplemental Table S1) indicated an enrichment of proteins localized to the “plasma membrane” (*p *=* *1.53 × 10^−114^; Supplemental Table S3). Because we analyzed the samples in the mass spectrometer in data-dependent mode, without quantification, we are unable to comment on differences between genotypes or immune treatments. Therefore, any immune-triggered events must be corroborated experimentally. Multiple sequence alignments of peptides spanning −10 to +10 amino- and carboxyl-terminal to the modified lysines indicated very little consensus and no significant motifs (Supplemental Figure S2). Unlike other post-translational modifications, the ubiquitination reaction requires coordination between E1 activating, E2 conjugating, and E3 ligase enzymes (Vierstra, 2012). While it may be possible for individual E2–E3 pairs to exhibit residue-level specificity on their target proteins, data from multiple species suggest that surface-availability may be the only unifying feature of ubiquitinated residues (Danielsen et al., 2011).

We identified ubiquitinated peptides mapping to proteins from diverse families, including aquaporins, H^+^ and Ca^2+^ ATPases, remorins, several classes of transporters, cellulose synthases, and others (Supplemental Tables S1–S2). Comparison between our dataset and eight published Arabidopsis ubiquitome datasets, as well as manual inspection of the literature, revealed 268 novel ubiquitin targets (Supplemental Table S4). We noted that molecular function GO terms “protein modification” (*p *=* *1.79 × 10^−12^), “phosphorylation” (*p *=* *2.15 × 10^−26^), and “response to stimulus” (*p *=* *6.44 × 10^−21^) were particularly enriched in our dataset (Supplemental Table S3). Interestingly, we identified multiple ubiquitinated lysines on over 70 RKs representing diverse subgroups, including FLS2, EFR, CERK1, LORE, RLK7, SOBIR1/EVR, LIK1, RKL1, WAK1, WAK2, FER, ER, BAM1, BAM2, and others (Table 1). We also identified ubiquitination sites on more than 20 plasma membrane-associated cytoplasmic protein kinases from several subgroups (Table 1). Because analysis of tryptic peptides with ubiquitinated lysine residues enriched by anti-K-ε-GG does not allow for discrimination between mono- or poly-ubiquitination, it is likely that we have captured both degradative and nondegradative ubiquitination on these protein kinases. Given the broad interest in phosphorylation-based signal transduction and protein homeostasis, we expect this information will be valuable to the plant research community and look forward to future studies that explore the function of these ubiquitination events.

## Supplemental data

The following materials are available in the online version of this article.

**Supplemental Methods.** Methods used in this study.

**Supplemental Figure S1.** Ubiquitinated residues identified on BIK1.

**Supplemental Figure S2.** Consensus motif analysis of ubiquitinated lysines.

**Supplemental Table S1**. High-confidence peptides identified in multiple experiments.

**Supplemental Table S2.** Peptides identified in single experiments.

**Supplemental Table S3.** Gene ontology terms associated with proteins identified in this study.

**Supplemental Table S4.** Comparative analysis reveals 268 unique ubiquitin targets identified in this study.

## Supplementary Material

kiab023_Supplementary_Data

## Notes

F.L.H.M. and J.M. designed the research; L.E.G. and F.L.H.M. performed the experiments; L.E.G., P.D., K.E.D., F.L.H.M., and J.M. analyzed the data; C.Z., F.L.H.M., and J.M. supervised the work; J.M. wrote the letter with input from all authors.

The author responsible for distribution of materials integral to the findings presented in this article in accordance with the policy described in the Instructions for Authors (https://academic.oup.com/plphys/pages/general-instructions) is: Jacqueline Monaghan (jacqueline.monaghan@queensu.ca).
