# Supplementary material for: Large-scale identification of ubiquitination sites on membrane-associated proteins in Arabidopsis thaliana seedlings
Source: Plant Physiol. 2021 Jan 28;185(4):1483–8. doi: 10.1093/plphys/kiab023 (PMC8133621; doi:10.1093/plphys/kiab023)
Supplement: kiab023_Supplementary_Data [file kiab023_supplementary_data.zip › pp.01149.2020-s01.pdf]

Large-scale identification of ubiquitination sites on membrane-associated proteins in *Arabidopsis thaliana* seedlings

Lauren E. Grubb[1,2,+], Paul Derbyshire[2], Katherine E. Dunning[1], Cyril Zipfel[2,3], Frank L.H. Menke[2,*], and Jacqueline Monaghan[1,2,*]

[1] Department of Biology, Queen's University, Kingston, Canada

[2] The Sainsbury Laboratory, University of East Anglia, Norwich Research Park, Norwich, United Kingdom

[3] Department of Plant and Microbial Biology, Zurich-Basel Plant Science Center, University of Zurich, Zurich, Switzerland

[+] Current address: John Innes Centre, Norwich Research Park, Norwich, United Kingdom

*Corresponding authors: frank.menke@tsl.ac.uk
jacqueline.monaghan@queensu.ca

Supplementary Data Includes:

- Supplemental Methods
- List of Supplemental Tables
- Supplemental Figures
- Supplemental References

Supplemental Methods

The plant genotypes used in this study have been previously described (Monaghan et al., 2014). Twenty mg of seed from each genotype was surface-sterilized using chlorine gas and stratified at 4°C in the dark for 2 days. Seed was then transferred to 250 mL Erlenmeyer flasks containing 50 mL 0.5X Murashige and Skoog (MS) media supplemented with 0.05% sucrose, and shaken at 100 rpm at ambient temperature with a 10h photoperiod for 8 days. Seedlings were incubated with 50 µM MG-132 (Sigma Aldrich, UK) for 1 h with shaking, and then vacuum infiltrated with water (mock) or 1 µM elf18 peptide (EZ Biolabs, USA) for 10 min. Samples were flash frozen and ground to a coarse powder with a mortar and pestle in liquid N_2, then homogenized in urea lysis buffer (8 M urea, 50 mM Tris-HCl pH 8.0, 150 mM NaCl, 1 mM EDTA, 1 mM P9599 protease inhibitor (Sigma Alrich), 1 mM phenylmethylsulfonyl fluoride (PMSF), 50 µM PR-619 (Sigma Aldrich)) using equal volume-to-tissue ratio in a Potter tube for 10 min at 1,000 rpm. An aliquot of 1 mL was removed and kept for quick analysis of elf18-induced MAPK activation by immunoblot as previously described (Monaghan et al., 2014) (data not shown). The remaining homogenate was centrifuged at 5,856 x *g* for 1 h at 4°C. The supernatant was transferred to a polycarbonate tube and centrifuged at 110,000 x *g* for 1 h at 4°C. The pellet was resuspended in 2 mL of lysis buffer with PMSF, and protein concentration was determined using the Bradford Assay according to the manufacturer's instructions (BioRad). Three mg of protein in 2 mL lysis buffer was incubated with 5 mM Tris(2-carboxyethyl)phosphine (TCEP) at ambient temperature for 45 min. Iodoacetamide (10mM) was added, and samples were incubated in the dark for 30 min at ambient temperature. Finally, samples were diluted to 10 mL in 50 mM Tris-HCl pH 8.0 and digested with 30 µg of trypsin (Pierce, UK) at 30°C overnight.

Following trypsin digestion, samples were acidified with aliquots of 50% trifluoroacetic acid (TFA) to pH 3.0. Precipitate was removed by centrifugation at 1,600 x *g*, and the peptides in the supernatant were purified using 2.5 mL C18 silica reversed-phase chromatography Sep-Pak columns (Waters). Prior to use, the columns were equilibrated sequentially with 1 column volume (CV) MeOH, then 1 CV activation buffer (80% acetonitrile (ACN), 0.1% TFA), and then 5 CV of wash buffer (2% ACN, 0.1% TFA). Sample was added to the column and allowed to drip by gravity flow, collected, and applied to the column a second time before application of 5 CV

wash buffer. Trypsin-digested peptides were eluted in 2 CV elution buffer (40% ACN, 0.1% TFA) and lyophilized.

The PTMScan® Ubiquitin Remnant Motif K-ε-GG antibody (Cell Signaling Technologies, UK) was first crosslinked to the agarose beads by washing each tube 3 times with 1 mL of 100 mM sodium borate pH 9.0, with centrifugation at 2,000 x *g* between each wash. The beads were then resuspended in 20 mM dimethyl pimelimidate (DMP) crosslinker and 100 mM sodium borate, and incubated for 1 h at room temperature with end-over-end rotation. The beads were washed twice with 1 mL of 200 mM ethanolamine pH 8.0, resuspended in 200 mM ethanolamine, and incubated overnight at 4°C with end-over-end rotation.

Prior to immunoaffinity purification (IP), crosslinked beads were washed 3 times with 1.5 mL IAP buffer (50 mM MOPS pH 7.2, 10 mM sodium phosphate, 50 mM NaCl). Lyophilized peptide samples were resuspended in 1.5 mL IAP buffer, sonicated for 10 min, and centrifuged at 16,000 x *g* for 5 min. A volume of 1,300 µL of peptide sample was added to 300 µL of K-ε-GG beads in IAP buffer, and the remaining 200 µL was reserved as 'input' sample. The IP was conducted for 2 h at 4°C with end-over-end rotation, followed by centrifugation for 1 min at 2,000 x *g.* The supernatant was kept as the 'unbound' sample to test the efficiency of di-Gly enrichment. Beads were washed twice with 1.5 mL IAP buffer, then 3 times with high performance liquid chromatography (HPLC) grade water prior to elution with 100 µL of 0.15% TFA, and centrifugation 1 min at 2,000 x *g*. The supernatant was kept as the 'elution' sample for analysis by LC-MS/MS.

Input, unbound, and elution samples were partially purified using C18 Micro-Spin Stagetips columns (The Nest Group Inc.). The samples were acidified with 50% TFA to a pH of 3.0, and the columns were placed in 2 mL microcentrifuge tubes and equilibrated by washing 3 times with 200 µL MeOH and centrifuged at 135 x *g* for 30 s between each wash. Equilibration continued with 3 washes of 200 µL equilibration buffer (80% ACN, 0.1% TFA) and centrifugation for 2 min at 185 x *g*, followed by washing 6 times with wash buffer (2% ACN, 0.1% TFA). Peptide solutions were loaded twice into the equilibrated columns and centrifuged for 4 min at 240 x *g*. Columns were then washed 6 times with Wash Buffer as above, followed by tandem elutions in 150 µL elution buffer (40% ACN, 0.1% TFA).

To prepare for mass spectrometry, the input, unbound, and eluted samples were dehydrated using vacuum centrifugation (SpeedVac), and then resuspended in

2% ACN and 0.1% Formic Acid (FA), followed by vortexing and sonication for 10 min prior to a final centrifugation at 10,000 xg for 10 min. Samples were then loaded into a 96-well plate for analysis on an Orbitrap Fusion Mass Spectrometer (ThermoFisher).

LC-MS/MS analysis was performed as described in (Bender et al., 2017) with the following modifications. On the Orbitrap Fusion MS/MS spectra were triggered with data dependent acquisition method using ‘top 20’ and ‘most intense ion’ settings. Peak lists in Mascot generic file format (.mgf files) were generated from raw files by using the MSConvert package (Matrix Science). Peak lists were searched using Mascot server v.2.4.1 (Matrix Science) against TAIR database (version 10), a separate in-house constructs database, and an in-house contaminants database. Tryptic peptides with up to 2 possible mis-cleavages and charge states +2, +3, +4, were allowed in the search. The following modifications were included in the search: oxidized methionine, diGly on lysine as variable modification and carbamido-methylated cysteine as static modification. Data were searched with a monoisotopic precursor and fragment ions mass tolerance 10ppm and 0.6 Da respectively. Mascot results were combined in Scaffold v. 4 (Proteome Software) and exported in Excel (Microsoft Office).

List of Supplemental Tables

Supplemental Table S1: High-confidence peptides identified in multiple experiments.

Supplemental Table S2: Peptides identified in single experiments.

Supplemental Table S3: Gene ontology terms associated with proteins identified in this study.

Supplemental Table S4: Comparative analysis reveals 265 unique ubiquitin targets identified in this study.

Supplemental Figures

A

BIK1 peptide sequence	K-GG	pBIK1:BIK1-HA					
		Col-0		CPK28-OE		cpk28-1	
		mock	elf18	mock	elf18	mock	elf18
KSSSTVAAAQKTEGEILSSTPVK	K31		1				1
KSSSTVAAAQKTEGEILSSTPVK	K41		2				
SSSTVAAAQKTEGEILSSTPVK	K41	2	2	4		2	2
SFTFNELKLATR	K61	2	3	2	2	2	3
LLVYEFMQKGSLENHLFR	K155	1					
MASVAVQcLSFEPKSRPTMDQVVR	K337		1				
ALQQLQDNLGKPSQTNPVKDTK	K358	1	1				2
ALQQLQDNLGKPSQTNPVKDTK	K366	1	2			1	1
ALQQLQDNLGKPSQTNPVKDTKK	K366/K369		1				
LGFKTGTTK	K374					1	
FTQKPFGR	K388	1	4	1			3

B

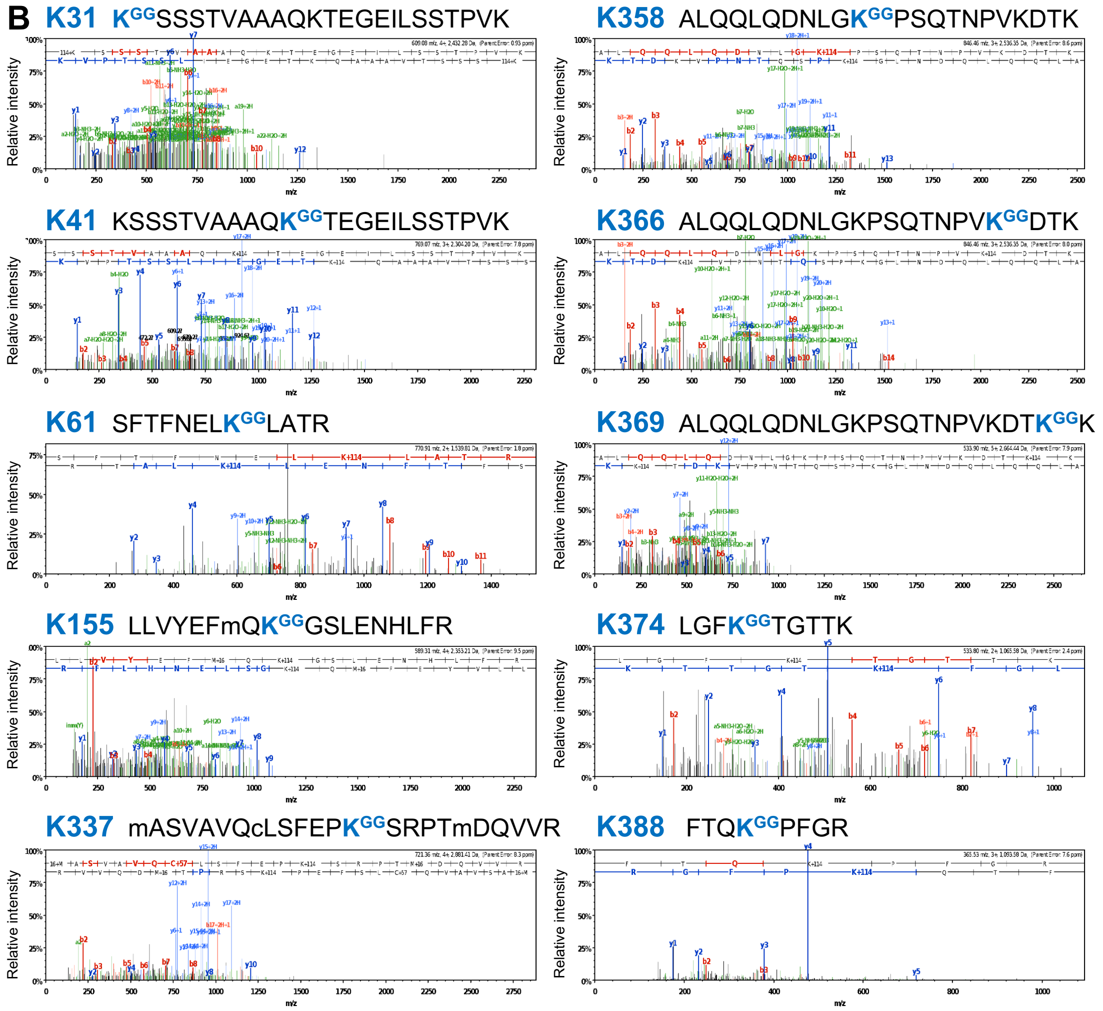

Supplemental Figure S1: Ubiquitinated residues identified on BIK1.

Ten tryptic peptides mapping to BIK1 contain di-Gly remnants in different genotypes and treatments as outlined in (A); the modified Lys residue is coloured blue. Mass spectra for each peptide were extracted from Scaffold and shown in (B); B- and Y-ions are coloured in red and blue, respectively.

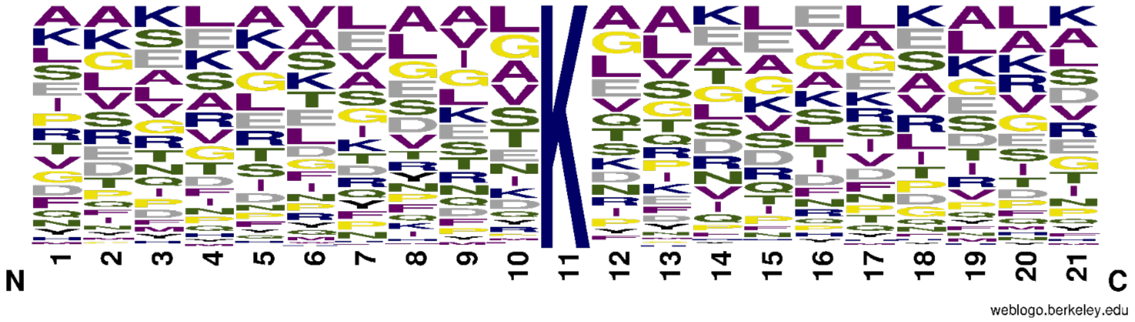

Supplemental Figure S2: Consensus motif analysis of ubiquitinated lysines.

Non-redundant full-length protein sequences were retrieved from The Arabidopsis Information Resource (TAIR) and peptide sequences -10 and +10 amino- and carboxyl-terminal to the modified lysines were identified using the MID function in Microsoft Excel. These 21-amino-acid peptide sequences were analyzed using the MEME Suite v5.1.1 MoMo motif finder tool (Bailey et al., 2009), however no statistically significant (p<0.05) consensus motifs were identified. A multiple-sequence alignment of the peptide sequences surrounding the modified lysine was created using WebLogo (Crooks et al., 2004). Blue residues contain positively-charged R-groups (KHR); Black residues are negatively-charged (DE); Magenta residues are hydrophobic (AILMFWYV); Green residues are polar uncharged (STNQ); Yellow residues are special cases (CGP).

Supplemental References

Bailey TL, Boden M, Buske FA, Frith M, Grant CE, Clementi L, Ren J, Li WW, Noble WS (2009) MEME SUITE: tools for motif discovery and searching. Nucleic Acids Res **37**: W202–8

Bender KW, Blackburn RK, Monaghan J, Derbyshire P, Menke FLH, Zipfel C, Goshe MB, Zielinski RE, Huber SC (2017) Autophosphorylation-based Calcium (Ca2+) Sensitivity Priming and Ca2+/Calmodulin Inhibition of Arabidopsis thaliana Ca2+-dependent Protein Kinase 28 (CPK28). J Biol Chem **292**: 3988–4002

Crooks GE, Hon G, Chandonia J-M, Brenner SE (2004) WebLogo: a sequence logo generator. Genome Res **14**: 1188–1190

Igawa T, Fujiwara M, Takahashi H, Sawasaki T, Endo Y, Seki M, Shinozaki K, Fukao Y, Yanagawa Y (2009) Isolation and identification of ubiquitin-related proteins from Arabidopsis seedlings. J Exp Bot **60**: 3067–3073

Johnson A, Vert G (2016) Unraveling K63 Polyubiquitination Networks by Sensor-Based

Proteomics. Plant Physiol **171**: 1808–1820

Kim D-Y, Scalf M, Smith LM, Vierstra RD (2013) Advanced proteomic analyses yield a deep catalog of ubiquitylation targets in Arabidopsis. Plant Cell **25**: 1523–1540

Manzano C, Abraham Z, López-Torrejón G, Del Pozo JC (2008) Identification of ubiquitinated proteins in Arabidopsis. Plant Mol Biol **68**: 145–158

Maor R, Jones A, Nühse TS, Studholme DJ, Peck SC, Shirasu K (2007) Multidimensional protein identification technology (MudPIT) analysis of ubiquitinated proteins in plants. Mol Cell Proteomics **6**: 601–610

Raudvere U, Kolberg L, Kuzmin I, Arak T, Adler P, Peterson H, Vilo J (2019) g:Profiler: a web server for functional enrichment analysis and conversions of gene lists (2019 update). Nucleic Acids Res **47**: W191–W198

Romero-Barrios N, Monachello D, Dolde U, Wong A, San Clemente H, Cayrel A, Johnson A, Lurin C, Vert G (2020) Advanced Cataloging of Lysine-63 Polyubiquitin Networks by Genomic, Interactome, and Sensor-Based Proteomic Analyses. Plant Cell **32**: 123–138

Svozil J, Hirsch-Hoffmann M, Dudler R, Gruissem W, Baerenfaller K (2014) Protein abundance changes and ubiquitylation targets identified after inhibition of the proteasome with syringolin A. Mol Cell Proteomics **13**: 1523–1536

Walton A, Stes E, Cybulski N, Van Bel M, Inigo S (2016) It's time for some "site"-seeing: novel tools to monitor the ubiquitin landscape in Arabidopsis thaliana. The Plant
